# Constitutive overexpression of TNF in BPSM1 mice causes iBALT and bone marrow nodular lymphocytic hyperplasia

**DOI:** 10.1111/imcb.12197

**Published:** 2018-09-08

**Authors:** Cyril Seillet, Elyas H Arvell, Derek Lacey, Michael D Stutz, Marc Pellegrini, Lachlan Whitehead, Joel Rimes, Edwin D Hawkins, Ben Roediger, Gabrielle T Belz, Philippe Bouillet

**Affiliations:** ^1^ The Walter and Eliza Hall Institute of Medical Research Parkville VIC 3052 Australia; ^2^ Department of Medical Biology The University of Melbourne Melbourne VIC 3010 Australia; ^3^ The Centenary Institute Camperdown NSW 2050 Australia

**Keywords:** Arthritis, BPSM1, heart valve disease, iBALT, IL‐17, IL‐23, NLH, nodular lymphoid hyperplasia, tertiary lymphoid organs, bronchus‐associated, TNF

## Abstract

BPSM1 (Bone phenotype spontaneous mutant 1) mice develop severe polyarthritis and heart valve disease as a result of a spontaneous mutation in the *Tnf* gene. In these mice, the insertion of a retrotransposon in the 3' untranslated region of *Tnf* causes a large increase in the expression of the cytokine. We have found that these mice also develop inducible bronchus‐associated lymphoid tissue (iBALT), as well as nodular lymphoid hyperplasia (NLH) in the bone marrow. Loss of TNFR1 prevents the development of both types of follicles, but deficiency of TNFR1 in the hematopoietic compartment only prevents the iBALT and not the NLH phenotype. We show that the development of arthritis and heart valve disease does not depend on the presence of the tertiary lymphoid tissues. Interestingly, while loss of IL‐17 or IL‐23 limits iBALT and NLH development to some extent, it has no effect on polyarthritis or heart valve disease in BPSM1 mice.

## Graphical abstract

Constitutive TNF overexpression predisposes mice to rheumatoid arthritis and heart valve disease, and causes development of tertiary lymphoid organs in the lungs and bone marrow. We show that the development of arthritis and heart valve disease does not depend on the presence of the tertiary lymphoid tissues, and that loss of IL‐17 or IL‐23 limits tertiary lymphoid organ development while having development no effect on polyarthritis or heart valve disease.

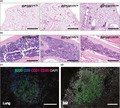

## Introduction

Tertiary lymphoid organs (TLO) such as bronchus‐associated lymphoid tissue (BALT) are follicular aggregates containing primarily B and T cells.^1^ Unlike secondary lymphoid organs such as Peyer's patches and nasal‐associated lymphoid tissue, the development of BALT is not preprogrammed and commences after birth. While BALT is common in rabbits and rats, it is virtually absent from healthy humans and mice.^1^ BALT is often found in the peri‐vascular space next to bronchi, but it can also develop in small airspaces that are apparently not adjacent to an artery or an airway. BALT can be induced in a number of pathological situations such as chronic inflammation, infection or autoimmunity, and for this reason is often referred to as inducible BALT (iBALT). While some of these clusters are only poorly organized aggregates containing mostly B cells, others have a complex structure containing B cells, T cells, follicular dendritic cells and specialized stroma, reminiscent of the lymphoid follicles found in secondary lymphoid organs. While iBALT appears to be the most common TLO, lymphocytic nodules have also been described in the joints of rheumatoid arthritis patients and in the salivary glands of Sjögren's syndrome patients.^2^ The presence of lymphocyte nodules in the bone marrow has been associated with systemic autoimmune diseases, chronic myeloproliferative disorders, and viral infection, and it can be benign or neoplastic. The causes underlying TLO development have not been completely elucidated but a number of chemokines (CXCL13, CCL19, CCL21) and their receptors (CXCR5, CCR7) have been implicated in the development of iBALT.^1^

In BPSM1 (Bone phenotype spontaneous mutant 1) mice, the insertion of a retrotransposon into the 3' unstranslated region (3' UTR) of the *Tnf* gene results in the generation of a mRNA lacking several motifs that are responsible for the posttranscriptional control of its stability.^3^ This mutation is dominant and causes *Tnf* mRNA to accumulate. As a result, TNF protein is overexpressed and found at very high levels in the serum of these mice, which develop severe polyarthritis and heart valve disease.^3^ Interestingly, iBALT is present in the lungs of all BPSM1 mice two weeks after birth and persists throughout their life. Similarly, all BPSM1 mice have benign lymphoid nodules in the bone marrow, first observed between four (*BPSM1* ^*m/m*^) and six (*BPSM1* ^*+/m*^) weeks after birth. No TLO was observed in the joints or salivary glands of BPSM1 mice. We have generated a number of compound mutant mice on the BPSM1 background, and report here the consequences of these additional mutations on the development of TLO. Remarkably, the development of polyarthritis and heart valve disease appears to be entirely independent of the presence of TLO.

## Results

### BPSM1 mice develop iBALT and nodular lymphoid hyperplasia

**Figure 1:**
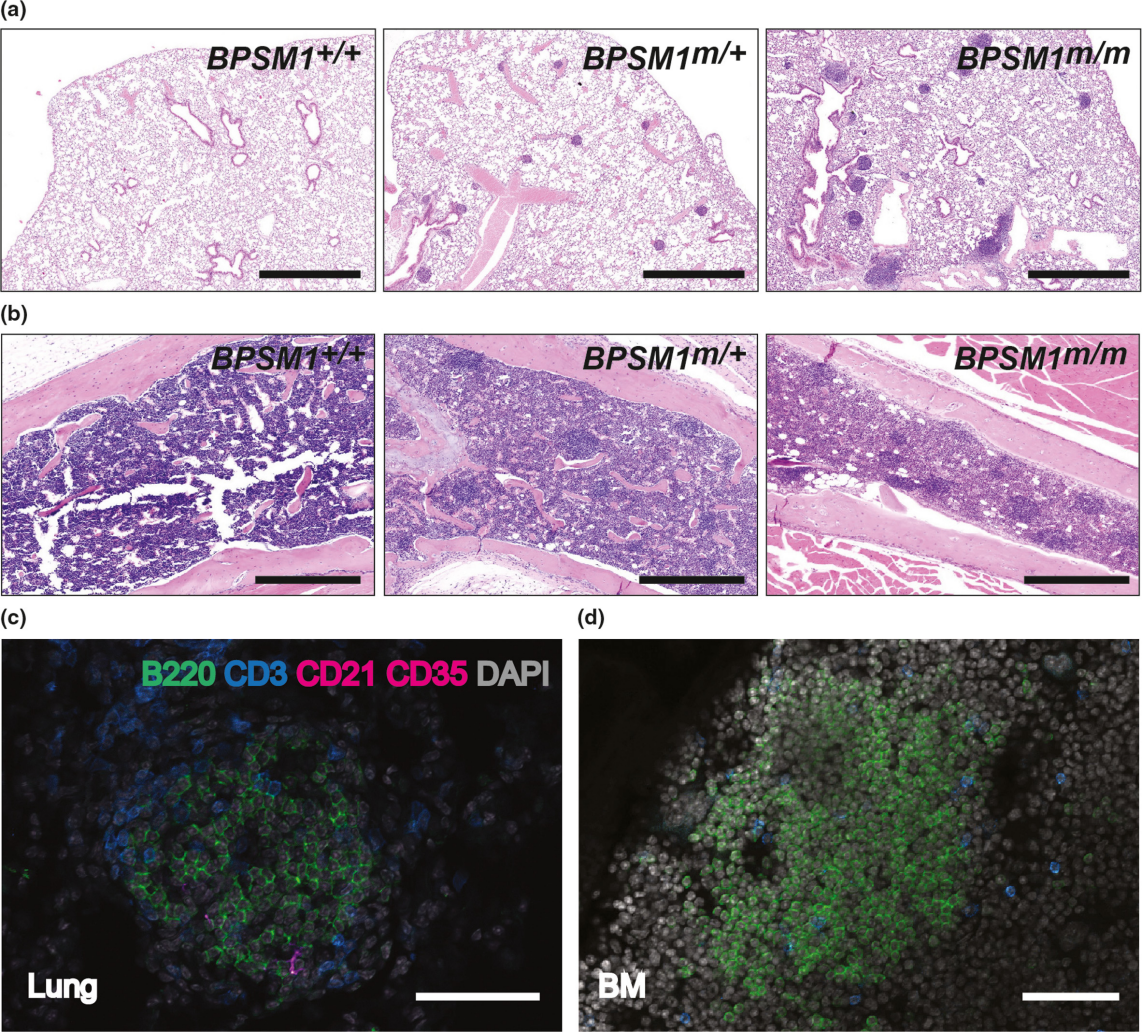
Spontaneous iBALT and NLH in BPSM1 mice. **(a)** Lung tissue stained with Hematoxylin and Eosin (H&E) showing iBALT in *BPSM1* ^*m/+*^ and *BPSM1* ^*m/m*^ mice. Scale bars, 1 mm. **(b)** H&E‐stained femoral bone marrow showing spontaneous nodular lymphoid hyperplasia in *BPSM1* ^*m/+*^ and *BPSM1* ^*m/m*^ mice. Scale bars, 0.5 mm. **(c)** Confocal image showing the presence B cells (B220), T cells (CD3) and follicular dendritic cells (CD21/CD35) in iBALT of *BPSM1* ^*m/+*^ mice. Scale bar, 50 μm. **(d)** Confocal image showing the presence of B cells and T cells in bone marrow lymphoid nodules of *BPSM1* ^*m/+*^ mice. Scale bar, 50 μm.

BPSM1 mice have a mutation in the *Tnf* gene causing constitutive overproduction of TNF, particularly from monocyte/macrophage populations.^3^ Heterozygous *BPSM1* ^*m/+*^ animals show the first overt symptoms of arthritis around 10 weeks of age. They put on weight more slowly than their wildtype (WT) littermates, and their arthritis gradually becomes more severe until they become paralyzed around 200 days. Homozygote *BPSM1* ^*m/m*^ animals display clear signs of severe arthritis by 2 weeks of age and become paralyzed between 4 and 6 weeks after birth. Histological analysis also revealed the presence of valvular inflammation in the heart of these mice, a condition whose severity depends on the genetic background and proved fatal in BPSM1‐BALB/c mice.^3^ We noticed that the lungs of all affected BPSM1 mice contained lymphocyte aggregates not only in close proximity to major airways, but also in places quite distant from them (Figure 1a). These lymphocyte foci were not observed in the first 5 days after birth but were clearly present in *BPSM1* ^*m/+*^ mice at postnatal day 10, and were a distinctive feature of all mutant animals thereafter (not shown). The development of iBALT in BPSM1 mice distinctly preceded the development of arthritis or heart disease symptoms. Homozygote *BPSM1* ^*m/m*^ animals had significantly higher levels of circulating TNF than *BPSM1* ^*m/+*^ littermates, and their arthritis and heart disease was much more severe.^3^ In the lungs, lymphocyte foci were larger in size in *BPSM1* ^*m/m*^ animals than in *BPSM1* ^*m/+*^ animals, but not more numerous (Figure 1a, see also Figure 3).

Distinctive lymphocyte nodules were also present in the bone marrow of all BPSM1 mice. These nodules appeared around 6 weeks after birth in heterozygote *BPSM1* ^*m/+*^ animals. In homozygote *BPSM1* ^*m/m*^ animals, the nodules appeared around 4 weeks after birth and were more numerous than in *BPSM1* ^*m/+*^ animals (Figure 1b, see also Figure 3). Immunohistochemical analysis showed that these lung and bone marrow follicles contained predominantly B cells, a feature quite common in iBALT,^1^ but that T cells were also present (Figure 1c, d). In addition, follicular dendritic cells were associated with iBALT but absent from the bone marrow nodules (Figure 1c, d). Nodular lymphoid hyperplasia (NLH) in the bone marrow has been reported in human patients and is associated with various chronic inflammatory conditions and blood neoplasms.^4^, ^5^, ^6^ In contrast, there are very few reports of these benign lymphoid follicles in mouse bone marrow. Interestingly, while iBALT was present in CCR7‐deficient mice as reported,^7^ no bone marrow nodules were present in these animals (Supplementary figure 1a).

Development of TLO in the salivary glands and arthritic joints have been reported to be associated with chronic inflammation.^2^ However, we never observed TLO in either of these locations in *BPSM1* ^*m/+*^ or *BPSM1* ^*m/m*^ mice, even at a very advanced stage of disease (not shown).

We conclude that BPSM1 mice develop lymphocytic aggregates in the lungs and bone marrow, and that the size of these aggregates appears to be related to the level of serum TNF present in these mice and their age (Figures 1, 3).

### iBALT and NLH require the presence of B cells, but not T cells

**Figure 2:**
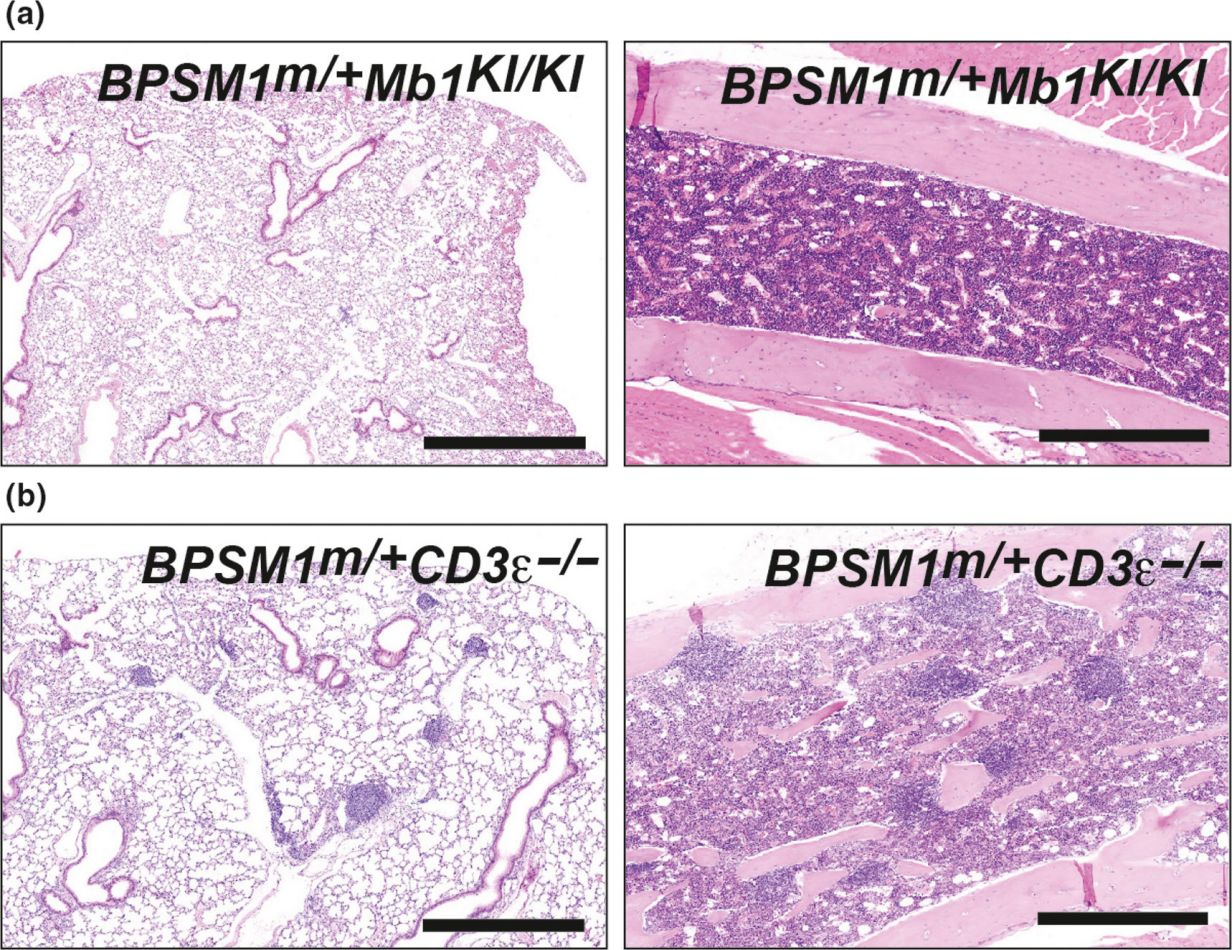
Loss of B cells prevents iBALT and NLH, loss of T cells does not. **(a)** H&E‐stained lung tissue (left hand panels) and femoral bone marrow (right hand panels) from a) *BPSM1* ^*m/+*^ *Mb1* ^*Ki/Ki*^ and **(b)** *BPSM1* ^*m/+*^ *CD3*ε^*−/−*^ mice. Scale bars, 1 mm for lung panels, 0.5 mm for bone marrow panels.

Certain models of polyarthritis clearly require the presence of B and/or T cells as drivers of the pathology. In order to determine the role of B and T cells in the pathology of BPSM1 mice, we crossed them with Mb1‐cre knockin^8^ and CD3ε knockout^9^ mice, respectively. The absence of B cells (due to the homozygous knockin of the *Mb1‐cre* allele) or T cells (due to the loss of CD3ε) did not change the kinetics or severity of arthritis and heart disease in BPSM1 mice.^3^ In contrast, loss of B cells prevented the development of TLO in both the lungs and the bone marrow of *BPSM1* ^*m/+*^ *Mb1‐cre* ^*KI/KI*^ mice (Figure 2a), whereas lymphocyte follicles were still present in both these locations in T cell‐deficient animals (in *BPSM1* ^*m/+*^ *CD3*ε^*−/−*^ mice) (Figure 2b). This is consistent with the fact that B cells are the prominent cell type in iBALT and NLH and indicates that T cells are not required for the organization of these structures. Nevertheless, T cells likely participate in generating a robust immune response in iBALT.^1^, ^2^ Arthritis and heart disease developed independently from the presence of B cells in *BPSM1* ^*m/+*^ *Mb1‐cre* ^*KI/KI*^ animals, indicating that iBALT and NLH play no part in these diseases in the BPSM1 model.

We have previously reported that loss of Myd88 or GM‐CSF did not influence the development of arthritis in BPSM1 mice.^3^ Similarly, no difference in iBALT and NLH was noticed in *BPSM1* ^*m/+*^ *Myd88* ^*−/−*^ and *BPSM1* ^*m/+*^ *GMCSF* ^*−/−*^ animals (Supplementary figure 1b, c). In contrast, loss of TNFR1 was sufficient to completely prevent development of arthritis and heart disease in BPSM1 mice. Indeed, even homozygote *BPSM1* ^*m/m*^ animals had no sign of these diseases in the absence of TNFR1 at 1 year of age, despite having very high levels of TNF in their blood.^3^ Likewise, iBALT and NLH were completely absent in these animals (Supplementary figure 1d), indicating that TNFR1, but not TNFR2, is required for the development of TLO in BPSM1 mice.

### TNFR1 is required for iBALT and NLH development

To further dissect the requirement of TNFR1 in the establishment of TLO, arthritis and heart disease, we performed bone marrow reconstitution experiments. All WT C57BL/6 Ly5.1 recipient mice were lethally irradiated (2× 5.5 Gy) at 6 weeks of age, then injected with 2 × 10^6^ bone marrow cells of the indicated genotypes and examined 5 months posttransplant unless otherwise indicated. *BPSM1* ^*m/+*^ recipients were irradiated at 4 weeks of age before they developed any sign of arthritis and injected with 2 × 10^6^ WT bone marrow cells.

WT mice that received *BPSM1* ^*m/+*^ bone marrow failed to develop iBALT but had bone marrow nodules that were larger than those of *BPSM1* ^*m/+*^ mice of the corresponding age. Additionally, these mice developed arthritis and heart valve disease (Table 1 and Supplementary figure 2a, b). These results suggest that the niche that allows iBALT development in *BPSM1* ^*m/+*^ mice is established early postnatally in response to high TNF levels, but that high TNF levels are not sufficient to establish the niche in older mice. In contrast, *BPSM1* ^*m/+*^ bone marrow alone was sufficient for the development of NLH. WT recipients of *BPSM1* ^*m/m*^ bone marrow also did not develop iBALT, although rare lymphocyte aggregates could sometimes be observed loosely attached to major airways (Supplementary figure 2c). These mice developed numerous large lymphocyte follicles in their bone marrow (Supplementary figure 2d). It is important to note that, because of severe arthritis, these animals could not be examined any later than two months posttransplant. It is therefore possible that the few aggregates observed in the lungs of *BPSM1* ^*m/m*^ into WT chimeric mice would have developed into more well‐organized iBALT if the reconstitution had lasted 5 months.

**Table 1:** Summary of the results of bone marrow reconstitution experiments

Donor/Recipient	iBALT	BM NLH	Arthritis	HVD
*BPSM1* ^*m/+*^ to *WT* (5 month)	NO	YES, increased	YES	YES
*WT to BPSM1* ^*m/+*^ (5 month)	YES	NO or very reduced	NO	NO
*BPSM1* ^*m/+*^ to *TNFR1* ^*−/−*^ (5 month)	NO	NO	NO	NO
*BPSM1* ^*m/m*^ *to WT* (2 month)	NO	YES	YES	YES
*BPSM1* ^*m/m*^ *TNFR1* ^*−/−*^ to WT (2 month)	NO	YES	YES	YES

The development of iBALT, bone marrow NLH, arthritis and heart valve disease (HVD) was examined following transplantation of 2 × 10^6^ donor bone marrow cells to lethally‐irradiated recipients. At least five recipients were examined in each transplantation experiment. Note that recipients of *BPSM1* ^*m/m*^ and *BPSM1* ^*m/m*^ *TNFR1* ^*−/−*^ bone marrow were analysed 2 months after transplant because of severe arthritis.

*BPSM1* ^*m/+*^ mice that received WT bone marrow still had iBALT 5 months after the transplant (Table 1 and Supplementary figure 2e, f), despite having normal TNF levels in their serum.^3^ In contrast, bone marrow nodules were rare or absent in these mice. These animals did not develop arthritis or heart valve disease. This indicates that the iBALT niche established early in *BPSM1* ^*m/+*^ animals is still active after irradiation and is also capable of attracting WT cells in the presence of normal levels of circulating TNF. The near‐complete absence of bone marrow lymphocytic nodules in these mice indicated the efficiency of the reconstitution, and the requirement of high levels of hematopoietically‐derived TNF for the development of NLH. Importantly, both these points were corroborated by the absence of arthritis and heart disease in *BPSM1* ^*m/+*^ recipients of WT bone marrow.

*TNFR1* ^*−/−*^ recipients of *BPSM1* ^*+/−*^ bone marrow had neither iBALT nor NLH (Table 1 and Supplementary figure 2g, h), and did not develop arthritis or heart disease. While no conclusion can be made about iBALT development from this experiment since WT recipients of *BPSM1* ^*m/+*^ bone marrow also had no iBALT, this result suggests a requirement for TNFR1 in non‐hematopoietic cells for the development of NLH, since the transplant of *BPSM1* ^*m/+*^ marrow to irradiated WT recipients led to large lymphocyte nodules in the bone marrow. It thus appears that the establishment of the niche that allows NLH development requires the participation of nonhematopoietic cells expressing TNFR1.

To determine whether TNFR1 was needed in the hematopoietic compartment for the development of iBALT and NLH, we transplanted the bone marrow of *BPSM1* ^*m/m*^ *TNFR1* ^*−/−*^ donors into irradiated WT recipients and examined the mice 2 months later (again, time‐limited because of the development of severe arthritis). Only a few poorly organized lymphocyte aggregates could be observed in these animals (Table 1 and Supplementary figure 2). In contrast, and despite the fact that *BPSM1* ^*m/m*^ *TNFR1* ^*−/−*^ donors themselves never develop NLH (Supplementary figure 1d), WT recipients of *BPSM1* ^*m/m*^ *TNFR1* ^*−/−*^ bone marrow had NLH (Supplementary figure 2j), demonstrating that overexpression of TNF triggers NLH by acting on a nonhematopoietic component, and that the expression of TNFR1 in the hematopoietic compartment is not necessary for NLH, arthritis or heart valve disease.

### IL‐23 and Il‐17 play a role in lymphoid neogenesis

**Figure 3:**
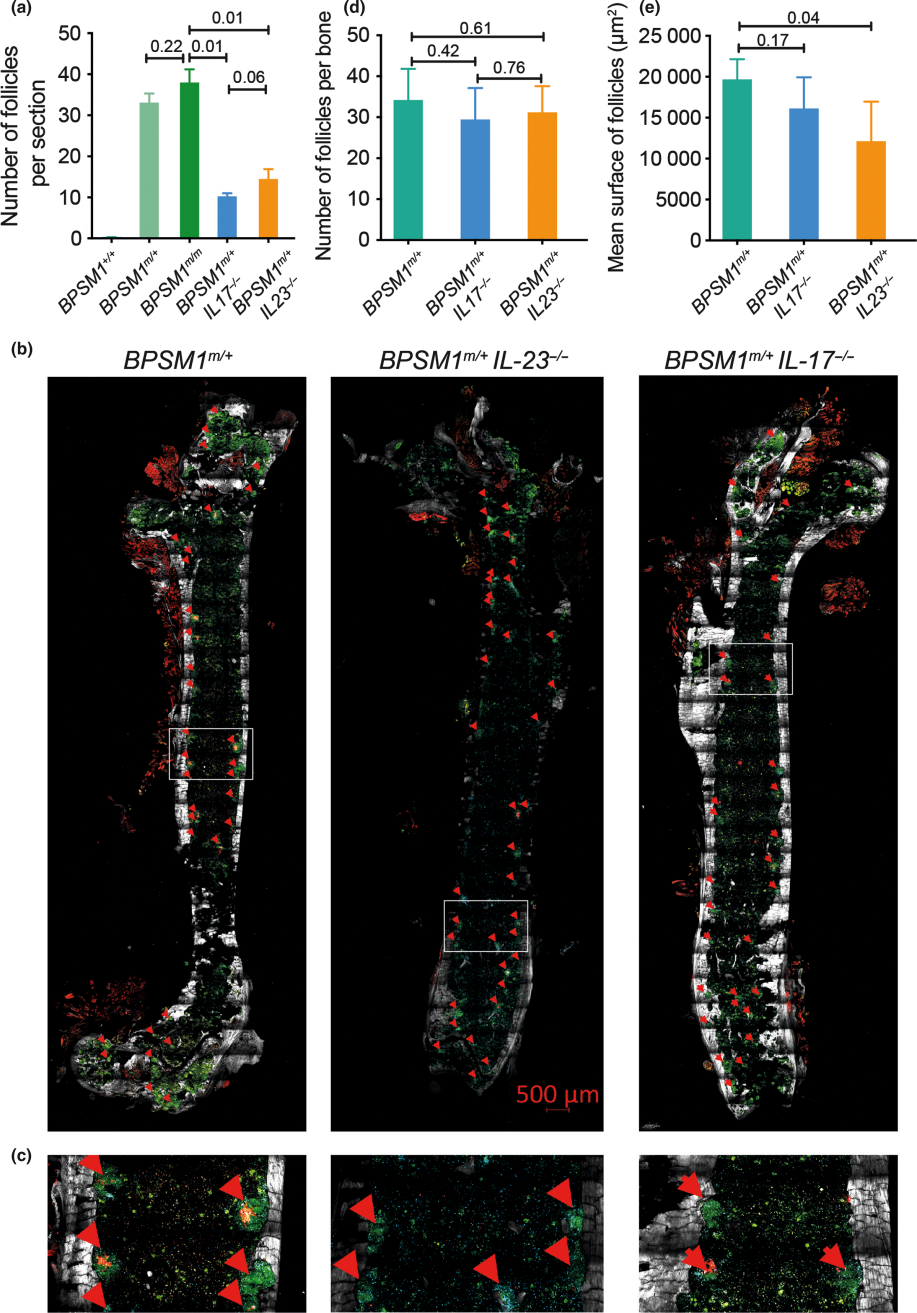
Loss of IL‐17 or IL‐23 influences iBALT and NLH formation in BPSM1 mice. **(a)** Quantification of iBALT follicles on H&E‐stained lung sections from *WT*, *BPSM1* ^*m/+*^, *BPSM1* ^*m/m*^, *BPSM1* ^*m/+*^ *IL‐23* ^*−/−*^ and *BPSM1* ^*m/+*^ *IL‐17* ^*−/−*^ animals (5 sections per animal, *n* ≥ 5 animals for each genotype, mean ± s.d.). **(b)** Dual confocal/2‐photon microscopy showing the presence B cells (B220, green), T cells (CD3, blue), collagen (grey) and follicular dendritic cells (CD21, CD35, red) in nondecalcified femoral bone marrow of *BPSM1* ^*m/+*^, *BPSM1* ^*m/+*^ *IL‐17* ^*−/−*^ and *BPSM1* ^*m/+*^ *IL‐23* ^*−/−*^ animals. Red arrows indicate lymphocyte follicles. **(c)** Magnified picture of the highlighted area in the white rectangle in **b**. **(d)** Total number of follicles/section in *BPSM1* ^*m/+*^, *BPSM1* ^*m/+*^ *IL‐17* ^*−/−*^ and *BPSM1* ^*m/+*^ *IL‐23* ^*−/−*^ animals as determined from 2 sections of 2 mice per genotype. **(e)** Mean surface of follicles was calculated by adding the surface of each iBALT detected and dividing by the number of follicles per section. *P*‐values were calculated using a two‐tailed Student's *t*‐test performed using Prism (GraphPad) to determine statistical significance.

The IL‐23/IL‐17 axis is involved in the pathogenesis of several inflammatory diseases such as psoriasis, rheumatoid arthritis (RA), ankylosing spondylitis and inflammatory bowel disease.^10^, ^11^ Moreover, IL‐17, and to a lesser extent IL‐23, have been shown to contribute to iBALT formation.^12^ To examine the roles of the Il‐23/IL‐17 axis in the different pathologies present in BPSM1 mice, we crossed them to IL‐23p19‐13 and IL‐17A‐deficient^14^ mice, respectively. Loss of either IL‐23 or IL‐17 reduced iBALT in *BPSM‐1* ^*m/+*^ mice, although a small number of iBALT foci were still present in both *BPSM1* ^*m/+*^ *IL‐23* ^*−/−*^ and *BPSM1* ^*m/+*^ *IL‐17* ^*−/−*^ animals (Figure 3; Supplementary figure 3a). To quantify this effect, we counted lymphocyte foci on 5 H&E‐stained lung sections of multiple (*n* ≥ 5 for each genotype) *WT*,* BPSM1* ^*m/+*^, *BPSM1* ^*m/m*^, *BPSM1* ^*m/+*^ *IL‐23* ^*−/−*^ and *BPSM1* ^*m/+*^ *IL‐17* ^*−/−*^ animals (Figure 3a). While the numbers of iBALT follicles were similar in *BPSM1* ^*m/m*^ and *BPSM1* ^*m/+*^ mice, these numbers were halved in *BPSM1* ^*m/+*^ *IL‐23* ^*−/−*^ and *BPSM1* ^*m/+*^ */IL‐17* ^*−/−*^ animals. To study the effects of IL‐23 and IL‐17 loss on the bone marrow lymphoid nodules in the *BPSM1* ^*m/+*^ mice, we used dual confocal/multiphoton imaging (Figure 3b, c). The number of BM lymphoid nodules was not reduced in the absence of either IL‐23 or IL‐17, but their size was significantly decreased in the absence of IL‐23 (Figure 3b–e).

Mutations in the IL‐23 receptor gene have been associated with the development of ankylosing spondylitis.^15^ BPSM1 mice are a model of ankylosing spondylitis since they show inflammation of their spine as well as their sacroiliac joints. In contrast to the marked effects in the lungs and bone marrow, loss of neither IL‐23 nor IL‐17 had any effect on the kinetics or the severity of polyarthritis and heart disease in BPSM1 mice (Supplementary figure 3c, d). This is in agreement with our previous observation that loss of T cells did not modify the course of the disease in BPSM1 mice.^3^

Altogether, these results suggest that the IL‐23/IL‐17 axis plays an important role in the development of iBALT and NLH in BPSM1 mice, but these cytokines have little to no influence on the development of polyarthritis and heart disease in these animals.

### High systemic TNF levels and accompanying iBALT in BPSM1 mice are associated with improved control of Mycobacterium tuberculosis pulmonary infection

**Figure 4:**
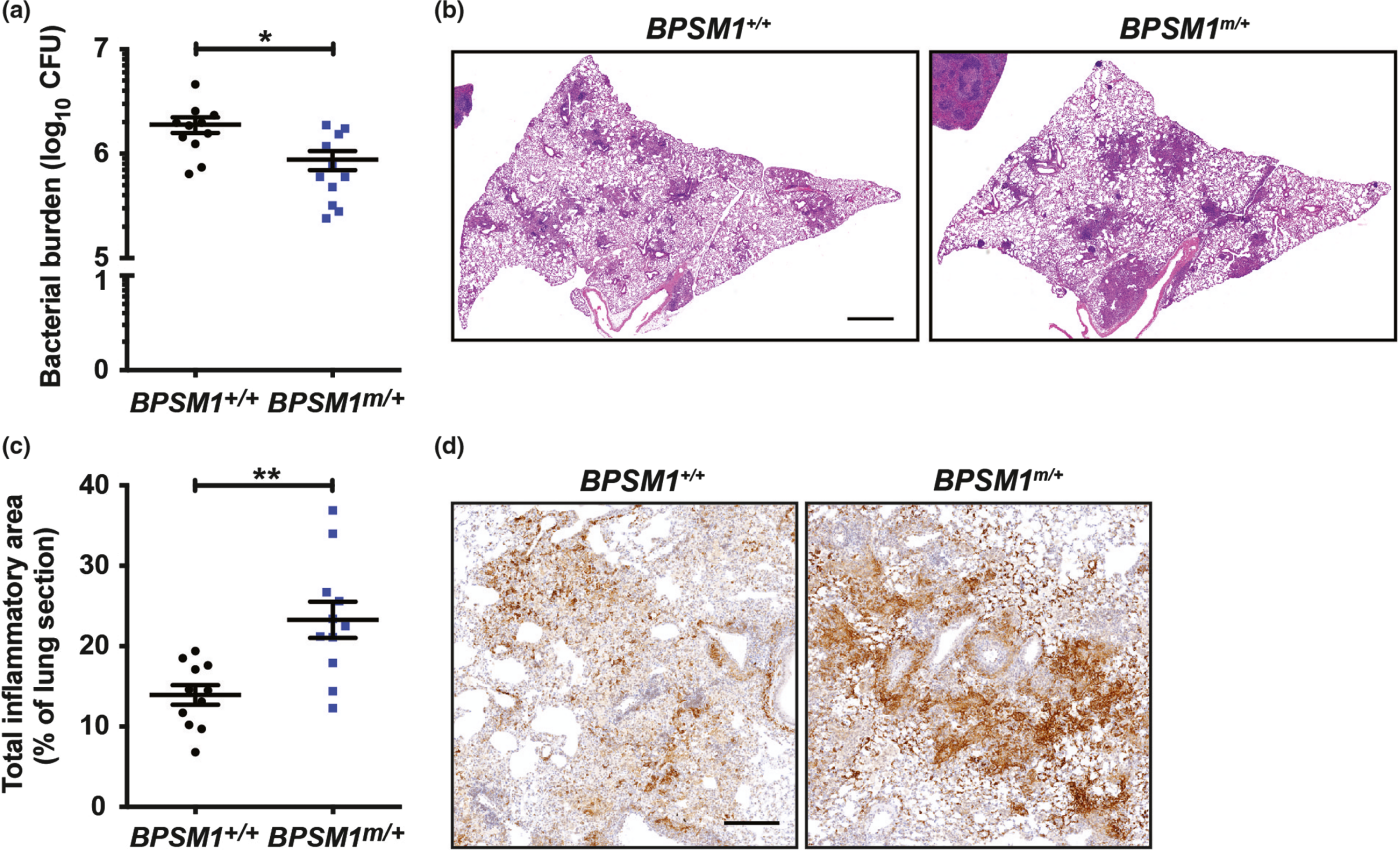
BPSM1 mice show improved ability to control M. tuberculosis infection. **(a)** Bacterial burdens in the lungs of *BPSM1* ^*+/+*^ and *BPSM1* ^*m/+*^ mice enumerated 5 weeks postinfection with aerosolized Mtb. **(b)** Lung histology of mice 5 weeks postinfection. Sections of the left lobe were stained with H&E. Scale bars, 1 mm. **(c)** Quantitation of the extent of pulmonary inflammation in H&E‐stained lung sections. **(d)** Immunohistochemical staining of F4/80 in lung inflammatory lesions. Scale bars, 0.2 mm. Graphs show mean and s.e.m. and each point represents one mouse (*n *=* *11 in each group). Data were analyzed by a Student's *t*‐test (*P < 0.05; **P < 0.01).

The elevated systemic levels of TNF in BPSM1 mice drives their overt phenotype including the development of iBALT. We speculated that the elevated levels of TNF in BPSM1 mice may also impact on the outcomes of infectious diseases. TNF is essential for the control of *Mycobacterium tuberculosis* (Mtb) in humans and mice.^16^, ^17^ Deficiency in TNF signaling causes rapid tuberculosis disease progression. However, it has been postulated that excessive TNF may equally promote Mtb disease pathogenesis by causing collateral tissue damage.^18^ To examine the impact of high systemic TNF levels, we infected *BPSM1* ^*m/+*^ and control animals with Mtb. We found that the high TNF levels, and accompanying lung phenotype, enhanced Mtb control and reduced lung bacterial loads in *BPSM1* ^*m/+*^ mice compared to littermate controls (Figure 4a). Lung histology showed a mild increase in pulmonary inflammation (Figure 4b, c), which correlated with an enrichment of F4/80^+^ macrophages within inflammatory lesions (Figure 4d). These data indicate that systemic elevations in TNF do not promote Mtb disease, as has been suggested by others,^18^, ^19^ but rather they enhance pulmonary clearance of the organism, perhaps in part by stimulating a beneficial macrophage response.

## Discussion

iBALT is a normal feature of certain mammals (e.g. rabbits and rats) while it is absent from healthy mice and humans. In humans and mice, the formation of iBALT appears intimately linked to chronic inflammation. The generation of disorganized lymphoid clusters in the lungs is easy to obtain in C57BL/6 mice upon intranasal administration of lipopolysaccharide (LPS).^12^ However, the formation of structured iBALT, containing many B cell follicles separated by well‐defined T cell areas, required that LPS be administered soon after birth. LPS administration to 2 or 3‐week‐old mice only produced disorganized B cell clusters lacking defined T cell areas.^12^ Thus, the competence of the mouse lungs to respond to high TNF levels and establish an iBALT niche occurs early after birth and is limited in time. BPSM1 mice overexpress TNF, but their levels of other inflammatory cytokines are normal.^3^ While it is tempting to speculate that TNF is involved in the response of the lung tissue to LPS, it is likely that other cytokines are also elevated in this model of BALT induction. Involvement of other cytokines may explain why it is relatively easy to induce iBALT with LPS when transplantation of *BPSM1* ^*m/+*^ or *BPSM1* ^*m/m*^ bone marrow could not. Of note, most of the conditions that induce the formation of BALT (such as response to Influenza virus, asthma, RA, Sjögren's syndrome, chronic obstructive pulmonary disease) involve a strong inflammatory response and multiple cytokines. Interestingly, *BPSM1* ^*m/+*^ recipients transplanted with WT bone marrow could develop iBALT and maintain it while having normal serum levels of TNF posttransplantation.^3^ This indicates that the iBALT niche already established at the time of irradiation survives the treatment and is capable of recruiting wildtype B, T and dendritic cells to maintain the tissue over long periods. This suggests that the niche is made of and maintained by either nonhematopoietic cells (maybe the functional equivalent of mesenchymal stromal organizer cells required for the development of secondary lymphoid organs,^20^ or radio‐resistant tissue‐resident hematopoietic cells. Interestingly, emerging evidence suggests that dendritic cells are likely to be important organizers of iBALT formation,^21^ and some dendritic cell populations have been shown to be radio‐resistant (e.g. Langerhans cells^22^).

The presence or absence of iBALT in different species may be the result of differences in the expression of the *Tnf* gene around or soon after birth. However, the time restriction most likely is not absolute, since properly organized iBALT is found in adults in association with chronic diseases such as RA or viral infections. In most cases, the destruction of lung tissue is extensive, and it is possible that the healing process brings the tissue back to a state similar to that found in the few days following birth.

At first glance, the situation in the bone marrow appears different, since the transplant of *BPSM1* ^*m/+*^ bone marrow into irradiated adult WT recipients readily triggers the development of lymphoid nodules in the bone marrow, more extensive in fact than in the original *BPSM1* ^*m/+*^ donors. However, this may also occur because of the severe injury caused to the recipient's bone marrow by irradiation, which most likely also triggers healing processes similar to those evoked earlier. It is interesting to note that expression of TNFR1 in a nonhematopoietic cell type (or a radio‐resistant hematopoietic cell type) in the host, but not in the donor bone marrow cells, is necessary for the development of the lymphoid nodules in the bone marrow. Once again, mesenchymal stromal organizer cells come to mind.^20^ TNFR2 does not compensate for the loss of TNFR1 in this instance and this may simply reflect the absence of TNFR2 on this cell type, rather than a difference in signaling between the two receptors.

Whether bone marrow lymphoid nodules perform any particular function remains elusive. They have been described in patients suffering from a variety of infectious or inflammatory diseases, as well as in patients with chronic lymphocytic leukemia.^4^, ^5^, ^6^ In all cases, the presence of BM follicles seems to be associated with high circulating TNF levels, even in the case of chronic lymphocytic leukemia. Since benign lymphoid follicles are not so rare in human patients,^23^, ^24^ it is somewhat puzzling that their presence in mice is barely ever mentioned in the literature. An interesting difference with iBALT is that BM nodules evidently require persistent high levels of TNF to be maintained, whereas iBALT does not. In that regard, iBALT could be considered as an optional secondary lymphoid organ that sometimes develops after birth.

Rangel‐Moreno *et al*.^12^ have described a role for IL‐17 and IL‐23 in the formation of iBALT in response to intranasal LPS administration. Since mutations of IL‐23 receptor have been associated with the development of ankylosing spondylitis in humans, we wanted to examine whether loss of IL‐23 or IL‐17 would also decrease the severity of arthritis and the inflammation of the heart valves in BPSM1 mice. However, loss of either of these cytokines had no effect on the extent of bone erosion or valve thickening. This once more highlights the independence between iBALT development and arthritis.

Whether iBALT is beneficial or detrimental to lung immunity remains controversial. Indeed, the extent of iBALT correlates with disease severity in patients with chronic obstructive pulmonary disease^25^ or RA.^26^ Conversely, iBALT is normally present in perfectly healthy rats and rabbits and has been shown to help clear influenza virus infection in mice devoid of secondary lymphoid organs.^27^ Of note, none of our mice harboring iBALT has ever shown symptoms associated with chronic obstructive pulmonary disease, indicating that iBALT alone is unlikely to be a driver of this disease. It is tempting to speculate that iBALT contributes to defense against pulmonary pathogens, based on the reduced Mtb burden observed in *BPSM1* ^*m/+*^ mice. However, this requires further investigation.

Since the presence or absence of iBALT does not modify the course and severity of arthritis and heart disease development in BPSM1 mice, we suggest that it plays no part in these diseases, and that the extent of iBALT in chronic obstructive pulmonary disease and RA patients merely represents the severity of the inflammation in these patients, and possibly the amount of TNF produced in their lungs. Likewise, the presence of lymphoid nodules in the bone marrow of BPSM1 mice appears more like a developmental accident because of persistently high levels of TNF and the presence of a TNFR1‐expressing cell type capable of organizing these structures rather than a consequence of the arthritis or the heart valve disease.

## Methods

### Mice

All animal experiments were conducted with the approval of the Animal Ethics Committee of the Walter and Eliza Hall Institute. BPSM1 mice were the result of a spontaneous mutation.^3^ The generation of mice devoid of CD3ε, Myd88, GM‐CSF, TNFR1, IL‐17, IL‐23, CCR7, as well as that of Mb1‐cre knockin mice has been described.^8^, ^9^, ^13^, ^14^, ^28^, ^29^, ^30^, ^31^ All mice were on the C57BL/6 genetic background. At least ten mice of each genotype were examined.

### Bone marrow reconstitution

To perform bone marrow reconstitution, recipient mice (all on a C57BL/6 genetic background) were lethally irradiated (2× 5.5  Gy) at 4–6 weeks of age, then injected with 2 × 10^6^ bone marrow cells of the indicated genotypes, and examined 5 months posttransplant, except for the recipients of *BPSM1* ^*m/m*^ *TNFR1* ^*−/−*^ bone marrow, which were analysed 2 months after transplant.

### Histology and immunochemistry

5‐mm‐thick sections of formalin‐fixed paraffin‐embedded tissue were stained with Haematoxylin and Eosin (H&E). Images shown are representative of 10 or more animals analysed per genotype. For immunohistochemistry, tissues were fixed in 4% paraformaldehyde and infiltrated with sucrose (increasing concentrations; 10%, 20% and 30%) before being embedded in optimal cutting temperature compound freezing media (Sakura Fineteck). Frozen sections of 12‐μm thickness were fixed in acetone for 10 min and blocked with phosphate‐buffered saline (PBS) containing 3% BSA and Fc block. Sections were stained with antibodies for the detection of B220 (RA3‐6B2), CD3 (145‐2C11) and CD21/35 (8C12) from BD Biosciences (Franklin Lakes, NJ, USA). Images were acquired on a LSM780 confocal microscope (Zeiss, Oberkochen, Germany).

### Immunofluorescence of undecalcified long bone sections

Immunofluorescence of undecalcified bone was performed as described previously.^32^ 20‐μm bone sections were cut using a Cryojane tape transfer system (Leica microsystems, Wetzlar, Germany). Antibodies used were as above. After washing in PBS, slides were incubated with secondary antibodies, counter‐stained with DAPI, and mounted with Prolong Diamond anti‐fade (Invitrogen, Waltham, MA, USA). Imaging of sections was performed on an upright Zeiss LSM 880 with the following lasers: Argon (458‐, 488‐ and 514‐nm), a diode‐pumped solid‐state 561‐nm laser and a Helium‐Neon 633‐nm, a tunable infrared multiphoton laser (Mai Tai DeepSee 690–1020 nm, Spectraphysics, Santa Clara, CA, USA). Dual confocal/multiphoton imaging was performed by combining nondescanned and internal detectors. Bone was detected using second harmonic generation with collagen excited at 840–860 nm. Signal was detected using 420–480 nm NDD detectors. Multi Z‐plane tilescans were captured and stitched using Zen software (Zeiss) and data analyzed using Fiji/ImageJ. Quantification was performed by researchers blinded from genotype of mice.

### *M. tuberculosis* infection

Mice were infected with 50‐100 colony forming units of Mtb strain H37Rv (Nicholas P West, University of Queensland, Australia) by aerosol, using an Inhalation Exposure System (Glas‐Col, Terre Haute, IN, USA). Mice were euthanised 5 weeks postinfection. Lungs were inflated by intratracheal infusion of PBS and were aseptically removed. Left lobes were fixed overnight at 4°C in 4% paraformaldehyde, while right lobes and spleens were homogenised with steel beads in PBS+0.05% Tween‐80. Homogenates were serially diluted and spread on Middlebook 7H11 agar (BD Biosciences) supplemented with 0.5% (v/v) glycerol and 10% (v/v) OADC supplements [50 g L^−1^ bovine serum albumin, 20 g L^−1^ dextrose, 0.04 g L^−1^ catalase and 0.5 g L^−1^ oleic acid (Sigma‐Aldrich, St Louis, MO, USA)]. Colonies were counted following incubation for 21 days at 37°C, and were expressed as colony forming units/organ. Fixed lungs were embedded in paraffin, sectioned (5‐μm) and stained with either haematoxylin and eosin (H&E), or immunohistochemically with rat anti‐mouse F4/80 (WEHI) using the automated Omnis EnVision G2 template (Dako, Glostrup, Denmark). Slides were scanned with a Pannoramic SCAN II scanner (3D Histech, Budapest, Hungary) and analyzed quantitatively with custom‐written macros using FIJI software.
