# Supplementary material for: Constitutive overexpression of TNF in BPSM1 mice causes iBALT and bone marrow nodular lymphocytic hyperplasia
Source: Immunol Cell Biol. 2018 Sep 8;97(1):29–38. doi: 10.1111/imcb.12197 (PMC6378607; doi:10.1111/imcb.12197)
Supplement: Supplementary file 1 [file IMCB-97-29-s001.pdf]

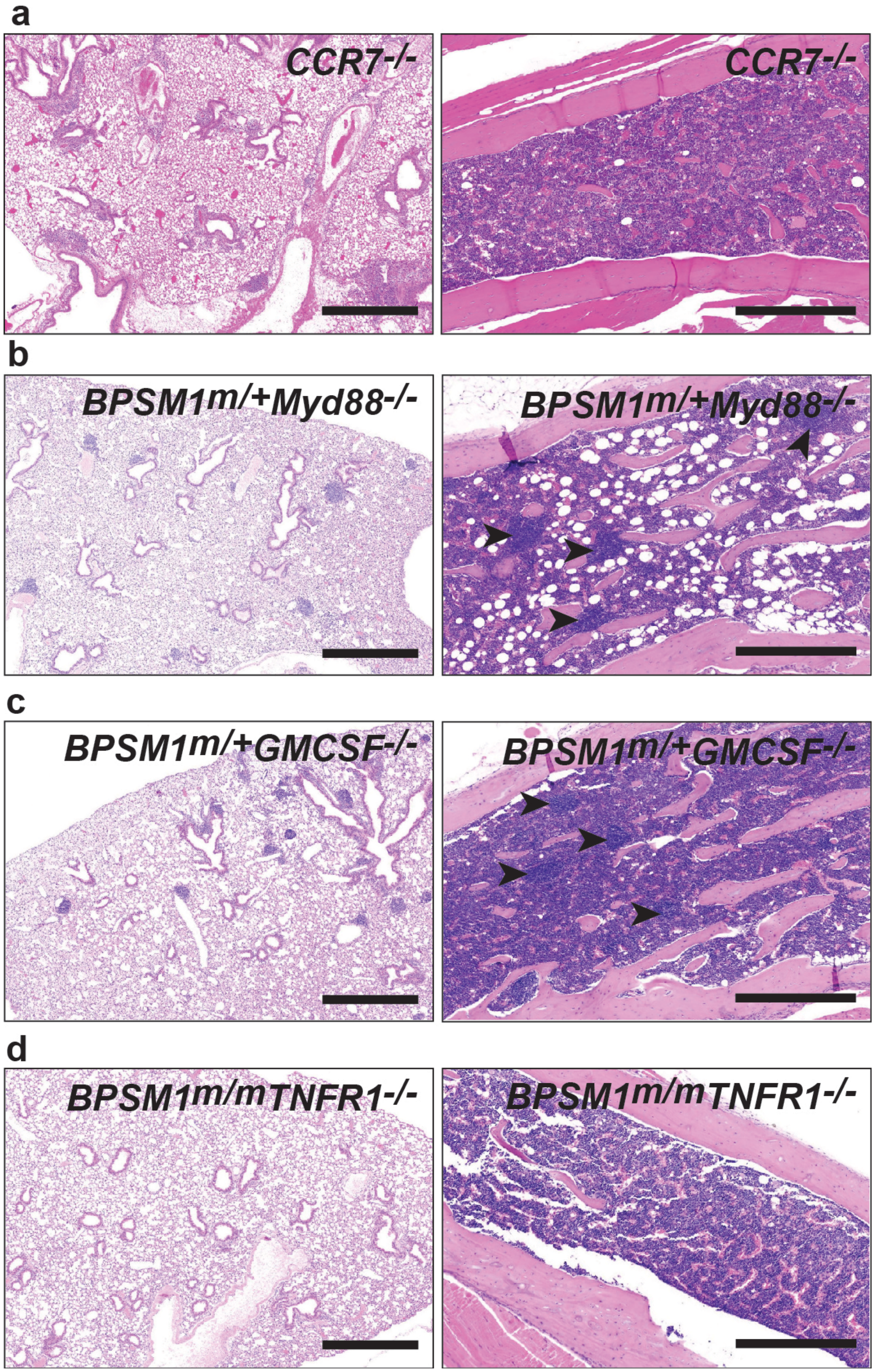

Supplementary figure 1

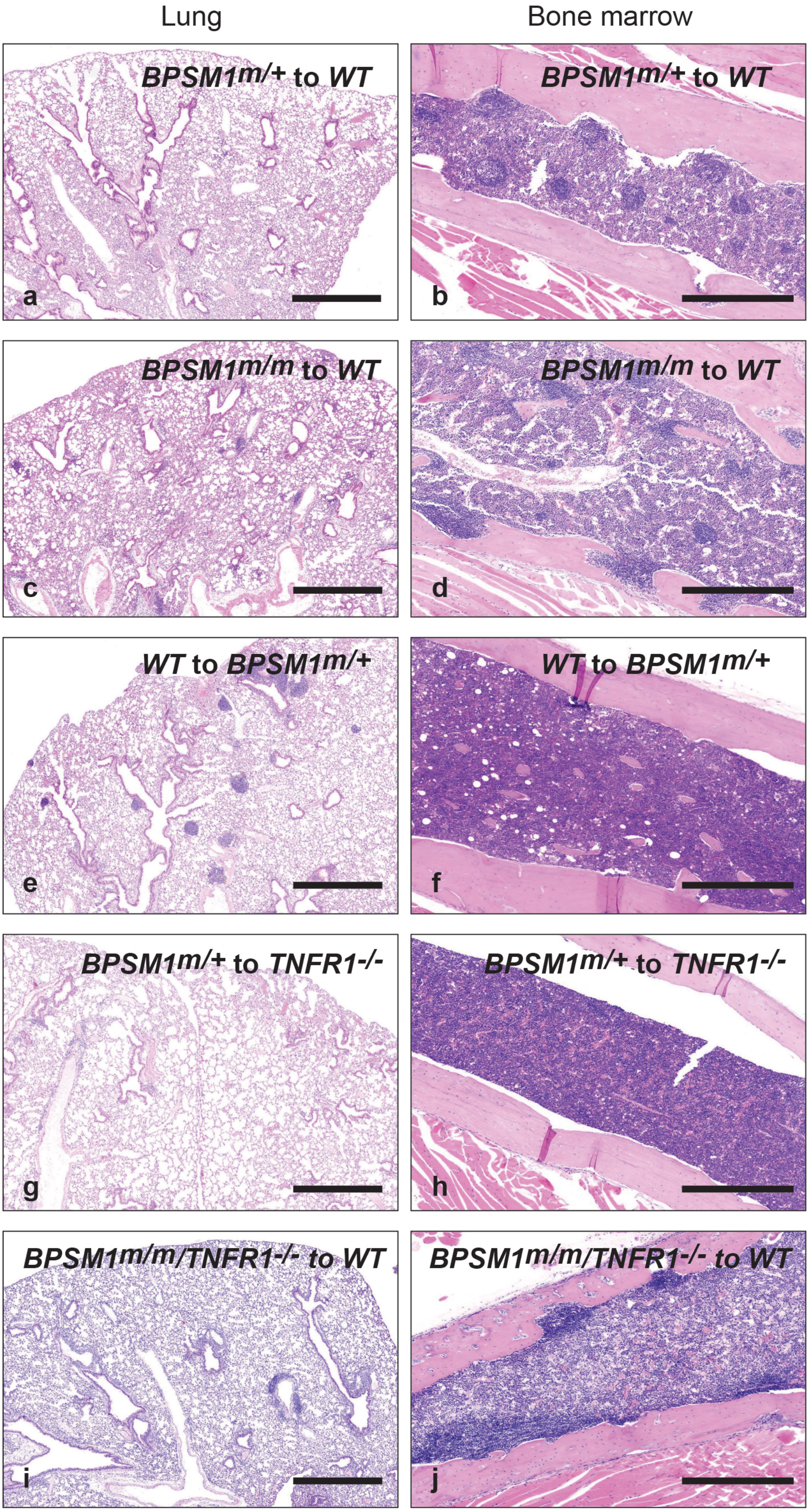

Supplementary figure 2

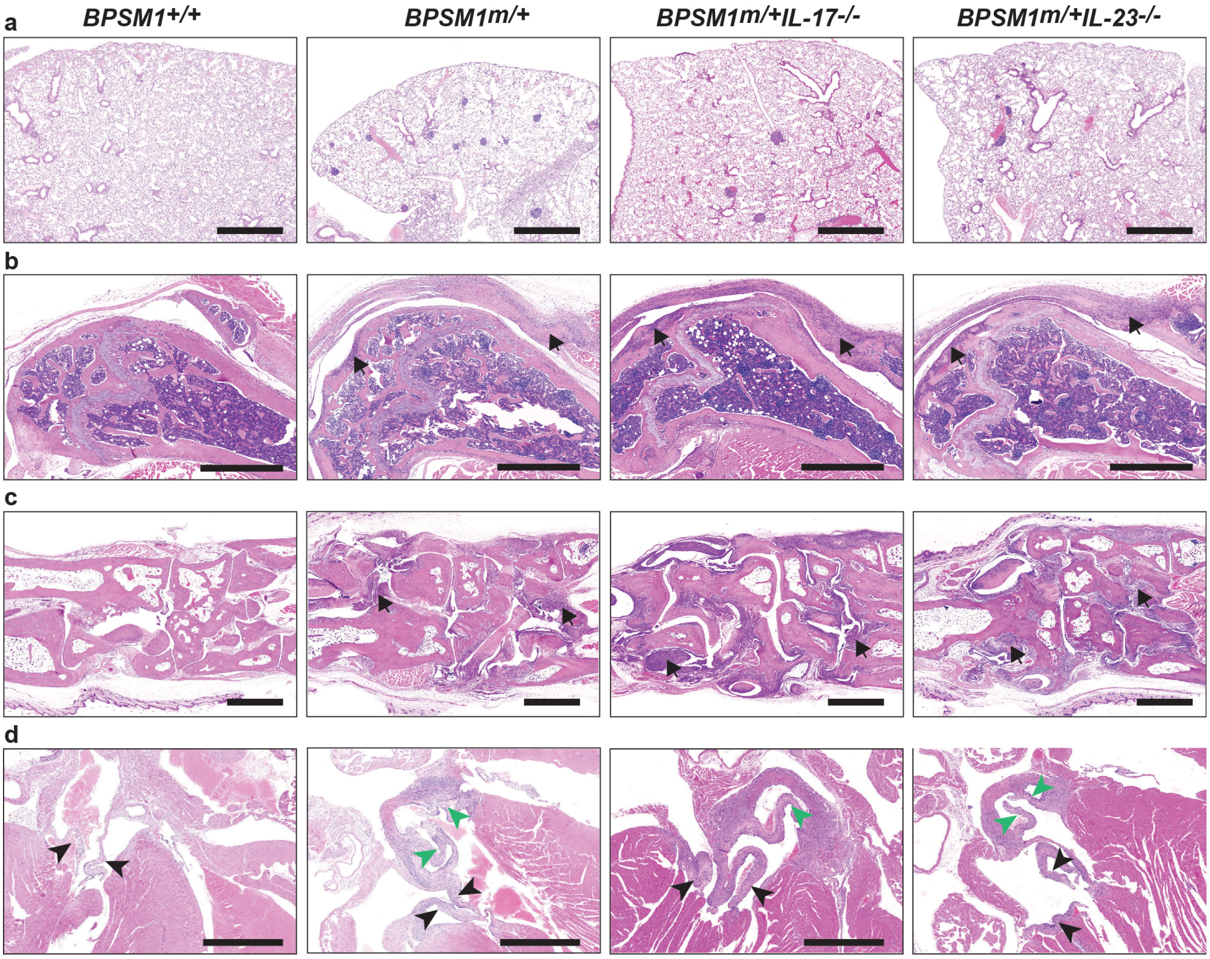

Supplementary figure 3

Legends to Supplementary figures

Supplementary Figure 1. ***iBALT and bone marrow NLH in the absence of CCR7, Myd88 or GM-CSF, or TNFR1.*** Representative H&E-stained lung (left panels) and bone marrow (right panels) sections from *CCR7*$^{-/-}$ (**a**), *BPSM1*$^{m/+}$*Myd88*$^{Ki/Ki}$ (**b**), *BPSM1*$^{m/+}$*GMCSF*$^{-/-}$ (**c**) and *BPSM1*$^{m/m}$*TNFR1*$^{-/-}$ mice (**d**). Arrowheads indicate bone marrow lymphocyte follicles. Scale bars, 1 mm (lung) or 0.5 mm (bone marrow).

Supplementary Figure 2. ***Bone marrow transplant experiments.*** H&E-stained lung tissue (**a**, **c**, **e**, **g**, **i**) and femoral bone marrow (**b**, **d**, **f**, **h**, **j**) from lethally-irradiated recipient mice transplanted with donor bone marrow. Genotypes of donors and recipients are indicated in each

panel. Scale bar, 1mm (**a**, **c**, **e**, **g**, **i**); scale bar, 0.5 mm (**b**, **d**, **f**, **h**, **j**). Results are described in text and summarised in Table 1.

Supplementary Figure 3. ***Loss of IL-17 or IL-23 has no impact on NLH, arthritis or heart valve disease.*** **a**) Representative H&E-stained lung tissue showing the presence of iBALT in *BPSM1*$^{m/+}$*IL-17*$^{-/-}$ and *BPSM1*$^{m/+}$*IL-23*$^{-/-}$ mice. **b**) Presence of NLH in the bone marrow of *BPSM1*$^{m/+}$*IL-17*$^{-/-}$ and *BPSM1*$^{m/+}$*IL-23*$^{-/-}$ mice. Note the extensive bone erosion and synovium hyperplasia in *BPSM1*$^{m/+}$*IL-17*$^{-/-}$ and *BPSM1*$^{m/+}$*IL-23*$^{-/-}$ animals (black arrows). **c**) The ankles of *BPSM1*$^{m/+}$*IL-17*$^{-/-}$ and *BPSM1*$^{m/+}$*IL-23*$^{-/-}$ mice show the typical bone erosion associated with severe arthritis (black arrows). **d**) Thickening of the mitral valves (black arrowheads) and inflammatory infiltration (green arrowheads) in the heart of *BPSM1*$^{m/+}$*IL-17*$^{-/-}$ and *BPSM1*$^{m/+}$*IL-23*$^{-/-}$. Scale bars, 1mm (**a**, **b**, **c**, **d**).
